# Transcriptome and targeted metabolome analysis reveals sugar metabolism regulation in maize stalks at different densities

**DOI:** 10.3389/fpls.2026.1784881

**Published:** 2026-05-08

**Authors:** Shuqi Ding, Huijuan Tian, Ying Hao, Wentao Du, Mengting Hu, Lei Zhang, Xifei Huang, Fangzhuo Sun, Jun Zhao, Jiajun Huang, Dan Zhang

**Affiliations:** 1 College of Agriculture, Tarim University, Alar, China; 2 Key Laboratory of Genetic Improvement and Efficient Production for Specialty Crops in Arid Southern Xinjiang of Xinjiang Corps, College of Agriculture, Tarim University, Alar, China

**Keywords:** maize stalk, sugar metabolism pathway, targeted metabolomics, transcriptome, WGCNA

## Abstract

The objective of this study is to investigate the regulatory mechanisms of sugar metabolism in maize stalks at varying planting densities. Targeted metabolomics and transcriptomics analyses were performed on stem samples of two varieties (M1 B12 and M2 J1590) at two planting densities (105,000 plants ha^-1^ and 135,000 plants ha^-1^) during the tasselling stage and grain filling stage. Combined with bioinformatics methods, these analyses elucidated sugar metabolism-related metabolites, gene expression patterns, and regulatory networks. Results showed that three structural components of the stalk accumulated significantly, sucrose content increased significantly, sucrose synthase activity decreased significantly, and sucrose phosphate synthase activity exhibited genotypic differences with increasing planting density and advancing growth stage. In targeted metabolomics, glucose was identified as the primary sugar metabolite. In transcriptome analysis, 39,757 differentially expressed genes were detected, whose expression was influenced by variety, density, and growth stage, and enriched in pathways such as hormone signal transduction, starch metabolism, and sucrose metabolism. In weighted gene co-expression network analysis, modules associated with monosaccharide metabolites were revealed, 12 sugar metabolism-related differentially expressed genes were identified, and a sugar metabolism regulatory network was constructed. These findings provide a theoretical basis for elucidating how maize stalk sugar metabolism responds to planting density. The screened candidate transcription factors offer indirect genetic resources for studying maize stalk lodging resistance mechanisms and references for improving lodging resistance in high-density maize planting.

## 1 Introduction

Maize (*Zea mays* L.) is one of the world’s most significant grains, playing a pivotal role in maintaining global food security (Qin et al., 2016). Planting density is one of the key agronomic measures influencing maize growth, development, and yield formation (Chen et al., 2012). However, an increase in planting density frequently results in intensified competition for light, nutrients, and water in the plant population, triggering adaptive changes in plant morphology and physiological metabolism (Shao et al., 2024). The regulatory mechanisms of stalk sugar metabolism are of particular importance (Li et al., 2019). The stalk, an essential component of the maize plant, fulfills two functions: It provides structural support for the aboveground parts and serves as a pivotal site for the storage and transportation of carbohydrates (S et al., 2014). As the primary components of the cell wall, cellulose, hemicellulose, and lignin are pivotal structural materials that support the plant’s resistance to lodging, and their synthesis is closely associated with sugar metabolism (Carpita et al., 1982; Liu et al., 2018). The process of accumulation and distribution of sugars in the stalk exerts a direct influence on various physiological processes, including grain filling, lodging resistance, and the utilisation value of biomass energy (Koch et al., 1996).

The process of sugar metabolism in maize stalks is subject to the coordinated regulation of multiple enzymes and genes, playing a crucial role in maize growth, development, and quality formation. Sucrose is one of the final products of plant photosynthesis, serving as the primary form of transport and distribution for photosynthetic products in higher plants, as well as a source of carbon and energy for the development of plant tissues and organs (Thomas et al., 1989). A substantial body of research has demonstrated that sucrose plays a pivotal role in the growth and development of plants (Xiao et al., 2024). In most maize varieties, increased planting density generally leads to a reduction in stalk sucrose content, accompanied by decreased activities of sucrose synthase (SS) and sucrose phosphate synthase (SPS), leading to inhibition of carbohydrate synthesis and accumulation. Key enzymes such as SS (Zhou et al., 1997; López-González et al., 2022), sucrose phosphate synthase (SPS) (Li et al., 2024), and invertase (Inv) (Bilska-Kos et al., 2020; Ma et al., 2020) act as “molecular switches,” thus regulating the synthesis, degradation, and distribution of sucrose with great precision.

The synthesis and activity of these enzymes are subject to the regulation of multiple genes. For instance, the proteins encoded by *Sh1* and *Sh2* are components of ADP-glucose pyrophosphorylase (AGPase), which influences the conversion of sucrose to starch and thereby indirectly affects stalk sugar metabolism (Yu et al., 2023; Saripalli and Gupta, 2015). Sucrose synthase (SUS) converts sucrose into fructose and uridine diphosphate glucose (UDP-glucose), which is used for the synthesis of cell wall polysaccharides together with uridine diphosphate (UDP) (Winter and Huber, 2000; Koch, 2004). Studies have shown that *IVR2* is expressed around the vascular bundles of stalks, which highly overlaps with the key sites of sucrose transport and hydrolysis (Y et al., 2000). These genes exhibit differential expression at different growth stages, which can dynamically regulate the accumulation of carbohydrates in stalks and maintain the balance of sugar metabolism.

## 2 Materials and methods

### 2.1 Experimental design and experimental materials

In accordance with the preceding research group’s identification of the mechanical traits of stalk lodging resistance, two maize inbred lines, designated M1 (B12) and M2 (J1590), were selected as experimental materials due to their significant phenotypic differences. The experiment was conducted on April 20, 2024 at the South Xinjiang Modern Agriculture Training Base for Industry-Education Integration of Tarim University. A randomized block design was employed, with two planting densities: The first experiment (D1) involved 105,000 plants ha^-1^, while the second experiment (D2) involved 135,000 plants ha^-1^. Each experimental material was replicated thrice under varying density conditions, with each plot arranged in a wide-narrow row planting configuration. Each plot spanned an area of 20 square meters. Two distinct growth stages were selected for the experimental materials: the tasseling stage (VT) and the grain-filling stage (R2). The selection of these stalks was made on the basis of three criteria: first, the presence of consistent growth patterns; second, the absence of any mechanical damage; third, the absence of pest or disease issues. The third internode at the base of the maize stalks was utilized as the experimental material. Following a rapid freezing process with liquid nitrogen, the materials were stored at -80 °C in an ultra-low temperature freezer for subsequent use. Each treatment was replicated thrice.

### 2.2 Determination of structural substance content in stalks

Lignin content (He et al., 2018): Lignin content was determined using a test kit from Suzhou Keming Company. The third internode at the base of the maize stalk was dried at 80 °C to a constant weight, crushed, and sieved through a 40-mesh screen, and approximately 5mg of the sample was weighed into a 10ml glass test tube. The sample was then processed according to the steps provided in the test kit. 1ml of the processed sample was taken into a quartz cuvette, and the absorbance value A at 280nm was measured. The experiment was set with 3 biological replicates and 3 technical replicates to ensure the reliability of the experimental results.

The correction formula for absorbance value:

(1) $$\text{ΔA}={\text{A}}_{1}-{\text{A}}_{0}$$

where A_1_ is the absorbance of the sample tube, and A_0_ is the absorbance of the blank tube.

The standard curve equation drawn in the experiment:

(2) $$\text{Y}=0.0694\text{x}+0.0068,\;{\text{R}}^{2}=0.9889$$

The calculation formula for lignin content (mg/g dry weight) is as follows:

(3) $$\text{Lignin}=\frac{\text{ΔA}-0.0068}{0.0694}\times \frac{\text{V}\times 10^{-3}\times \text{T}}{\text{W}}=0.0294\times \frac{(\text{ΔA}-0.0068)\times \text{T}}{\text{W}}$$

V is the total reaction volume (2.04mL); W is the sample mass (g); and T is the dilution factor. The coefficient 0.0294 was calculated as 2.04×10^-3^/0.0694≈0.0294.

Cellulose content (Hussain et al., 2020): Cellulose content was determined using a test kit from Suzhou Keming Company. Dry the third internode of the maize stalk base at 80 °C until constant weight, grind, sieve through a 40-mesh screen, weigh approximately 0.01g of the sample, add 1mL of 80% ethanol, incubate at 90 °C for 20 minutes, cool to room temperature, centrifuge at 8,000g at 25 °C for 10 minutes, and discard the supernatant. Add 1.5mL of 80% ethanol and acetone to the precipitate, wash once with each (vortex for about 2 minutes, centrifuge at 8,000g at 25 °C for 10 minutes, discard the supernatant), and dry the precipitate. The resulting material is the crude cell wall. Then, determine the cellulose content using the kit according to the manufacturer’s instructions. Place the supernatant to be tested in a 95 °C water bath for 10 minutes, cool it to room temperature, take 200μL of the supernatant into a 96-well microplate, and measure the absorbance at 620nm. Set 3 biological replicates and 3 technical replicates for the experiment.

The correction formula for absorbance:

(4) $$\text{ΔA}={\text{A}}_{1}-{\text{A}}_{0}$$

where A_1_ is the absorbance of the sample tube, and A_0_ is the absorbance of the blank tube.

The regression equation measured under standard conditions:

(5) Y=5.25x−0.0043

where x is the concentration of the standard substance in mg/mL, and y is the absorbance.

The calculation formula for cellulose content (mg/g dry weight):

(6) $$\text{Cellulose}=\frac{(\text{ΔA}+0.0043)\times {\text{V}}_{2}\times \text{T}}{5.25\times \text{W}}=4.76\times \frac{(\text{ΔA}+0.0043)\times \text{T}}{\text{W}}$$

where V_1_ = 0.15mL is the volume of the reaction solution, V_2_ =1.25mL is the volume of the extraction solution, W is the dry weight of the sample (g), and T = 20 is the dilution factor. The simplified coefficient 4.76 was derived from (V_2_/5.25)×T=(1.25/5.25)×20≈4.76.

Hemicellulose content (Hussain et al., 2020): Dry the third internode of the maize stalk base at 80 °C until constant weight, grind, sieve through a 40-mesh screen, weigh approximately 0.01g of the sample, add 1mL of 80% ethanol, incubate at 90 °C for 30 minutes, cool to room temperature, centrifuge at 8,000g at 25 °C for 10 minutes, discard the supernatant and retain the precipitate, then dry at 80 °C. Proceed with the operation using the reagents provided in the test kit, mix thoroughly, place in a 90 °C water bath for 5 minutes, cool naturally, centrifuge at 8,000g at 25 °C for 10 minutes, take 200μL of the supernatant into a 96-well plate, and measure the absorbance value A at 540nm. The experiment was set with 3 biological replicates and 3 technical replicates to ensure the reliability of the experimental results.

The correction formula for absorbance is:

(7) $$\text{ΔA}={\text{A}}_{1}{\text{-A}}_{0}$$

The regression equation measured under standard conditions:

(8) $$\text{Y}=0.206\text{x}+0.0609,\;{\text{R}}^{2}=0.9931$$

where x is the concentration of the standard substance (mg/mL) and y is the absorbance value.

The calculation formula for hemicellulose content (mg/g dry weight) is:

(9) $$\text{Hemicellulose}=\frac{(\text{ΔA}-0.0609)\times {\text{V}}_{\text{total},\;\text{sample}}}{0.2906\times \text{W}}=6.88\times \frac{\text{ΔA}-0.0609}{\text{W}}$$

where W is the sample mass (g, dry weight), and V_total, sample_=2mL is the total volume of the added extraction solution. The simplified coefficient 6.88 was derived from V_total, sample_/0.2906 = 2/0.2906 ≈ 6.88.

### 2.3 Determination of sucrose content and related enzyme activities

Sucrose content (Liu et al., 2016): Sucrose content was determined using an assay kit from Suzhou Keming Biotechnology. Approximately 0.1g sample taken from the third internode at the base of maize stalk was ground at room temperature, mixed with 1mL of extraction solution, and incubated in an 80 °C water bath for 10min. After cooling, the sample was processed following the kit instructions. The mixture was shaken thoroughly and decolorized in an 80 °C water bath for 60min. After cooling again, the sample was centrifuged at 4,000g at room temperature for 10min. The supernatant was collected, mixed with the reagents provided in the kit, and heated in a boiling water bath for 30min. After cooling, 200μL of the final solution was taken for measurement at a wavelength of 480nm. Set 3 biological replicates and 3 technical replicates for the experiment.

(10) $$\text{Sucrose}=\frac{{\text{C}}_{\text{standard}}\times {\text{V}}_{2}\times ({\text{A}}_{3}-{\text{A}}_{1})}{({\text{A}}_{2}-{\text{A}}_{1})\times \text{W}}=40\times \frac{{\text{A}}_{3}-{\text{A}}_{1}}{({\text{A}}_{2}-{\text{A}}_{1})\times \text{W}}$$

where A_1_, A_2_, and A_3_ are the absorbance values of the blank tube, standard tube, and sample tube, respectively; C_standard_=1 mg/mL is the concentration of the standard sucrose solution; V_1_ = 0.025mL is the volume of the loaded sample; V_2_ = 1mL is the total volume of the extraction solution; and W is the fresh weight of the sample (g). The simplified coefficient 40 was derived from C_standard_×V_2_/V_1_ = 1×1/0.025 = 40.

Sucrose synthase (SS) activity (Liu et al., 2016): Sucrose synthase (SS) activity was determined using an assay kit from Suzhou Keming Biotechnology. Approximately 0.1g samples were collected from the third internode at the base of maize stalks. The tissue was homogenized with 1mL extraction solution under ice-bath conditions, followed by centrifugation at 8,000g and 4 °C for 10 min. The supernatant was immediately collected, mixed with the reagents provided in the kit, and incubated in a boiling water bath for 30 min. After cooling, 200μL of the solution was taken to measure the absorbance at 480nm. Set 3 biological replicates and 3 technical replicates for the experiment.

(11) $$\text{SS activity}=\frac{{\text{C}}_{\text{Standard}}\times ({\text{A}}_{0}-{\text{A}}_{1})}{\text{T}\times \text{W}}=100\times \frac{{\text{A}}_{0}-{\text{A}}_{1}}{\text{W}}$$

where A_0_ and A_1_ are the absorbance values at 0min and 10min, respectively; C_standard_=1,000μg/mL is the concentration of the standard solution; V_1_ = 0.01mL is the volume of the sample added to the reaction system; V_2_ = 1mL is the total volume of the extraction solution; W is the fresh weight of the sample (g); and T = 10min is the total reaction time.

Sucrose phosphate synthase (SPS) activity (Liu et al., 2016): Sucrose phosphate synthase (SPS) activity was determined using an assay kit from Suzhou Keming Biotechnology. Approximately 0.1g samples were collected from the third internode at the base of maize stalks. The tissue was homogenized with 1mL extraction solution under ice-bath conditions, followed by centrifugation at 8,000g and 4 °C for 10min. The supernatant was immediately collected, mixed with the reagents provided in the kit, and incubated in a boiling water bath for 30min. After cooling, 200μL of the solution was taken to measure the absorbance at 480nm. Set 3 biological replicates and 3 technical replicates for the experiment.

(12) $$\text{SPS activity}=\frac{{\text{C}}_{\text{standard}}\times {\text{V}}_{2}\times ({\text{A}}_{0}-{\text{A}}_{1})}{\text{T}\times \text{W}}=100\times \frac{{\text{A}}_{0}-{\text{A}}_{1}}{\text{W}}$$

where A_0_ and A_1_ are the absorbance values at 0min and 10min, respectively; C_standard_=1,000μg/mL is the concentration of the standard solution; V_1_ = 0.01mL is the volume of the sample added to the reaction system; V_2_ = 1mL is the total volume of the extraction solution; W is the fresh weight of the sample (g); and T = 10min is the total reaction time.

Experimental materials of metabolomics analysis were selected from two densities of M1 and M2 varieties at two growth stages, specifically the third internode of the stalk base (Bingqin et al., 2023). Each treatment was replicated three times biologically. The centrifuge tubes used for sampling were pre-chilled and labeled (**Table 1**). Sampling was performed quickly, and the samples were immediately placed in a liquid nitrogen tank, rapidly frozen with liquid nitrogen, and stored at -80 °C after half an hour. The samples were transported to Wuhan Jinosec Technology Co., Ltd. using dry ice. Accurately weigh the required standard samples and dissolve them in water to prepare a 10mg/mL standard solution stock solution. Then, take an appropriate amount of the standard solution stock solution and mix it to prepare standard mixtures with maximum concentrations of 60μg/mL, 50μg/mL, or 40μg/mL. Prepare the series of standard samples required for instrument analysis according to the following concentration gradient. Take a clean chromatography vial, weigh an appropriate amount of polysaccharide sample, add 125μl of 72% sulfuric acid solution for acid hydrolysis, and incubate at 30 °C for 1 hour. Then add 1.35mL of water, mix with a vortex mixer, and heat at 121 °C for 2 hours. Adjust to neutrality with 0.5M sodium hydroxide, dilute appropriately, and transfer to the chromatography vial for analysis. The chromatography system used was the Thermo ICS 5000+ ion chromatography system (ICS 5000+, Thermo Fisher Scientific, USA), with an electrochemical detector for analyzing and detecting monosaccharide components. Chromeleon software was used to process the chromatography data. A Dionex™ CarboPac™ PA20 column (150×3.0mm, 10μm) was used, with an injection volume of 5μL. The mobile phases were as follows: Phase A (ultrapure water), Phase B (0.1M NaOH), and Phase C (0.1M NaOH containing 0.2M NaAc). The flow rate was 0.5mL/min, and the column temperature was maintained at 30 °C. The elution gradient procedure was set as below:

**Table 1:** Sample numbers for transcriptome sequencing of maize stalks.

Sample ID	Group ID	Sample description
1Z1, 2Z1, 3Z1	Z1	D1-VT-M1
1Z2, 2Z2, 3Z2	Z2	D2-VT-M1
1Z2, 2Z2, 3Z2	Z3	D1-R2-M1
1Z4, 2Z4, 3Z4	Z4	D2-R2-M1
1X1, 2X1, 3X1	X1	D1-VT-M2
1X2, 2X2, 3X2	X2	D2-VT-M2
1X3, 2X3, 3X3	X3	D1-R2-M2
1X4, 2X4, 3X4	X4	D2-R2-M2

0min: A/B/C = 95:5:0 (V/V); 26min: A/B/C = 85:5:10 (V/V); 42min: A/B/C = 85:5:10 (V/V); 42.1min: A/B/C = 60:0:40 (V/V); 52min: A/B/C = 60:40:0 (V/V); 52.1min: A/B/C = 95:5:0 (V/V); 60min: A/B/C = 95:5:0 (V/V).

Chromatographic data were processed using Chromeleon software.

The content of each component in solid samples (μg/mg) was calculated as:

(13) $$\text{Component content}=\frac{\text{C}\times \text{V}\times \text{F}}{\text{M}}$$

where C is the concentration detected by the instrument (μg/mL), V is the total volume of the sample extraction solution (mL), F is the dilution factor, and M is the fresh weight of the sample (mg).

### 2.4 Transcriptome sequencing and analysis

Twenty-four cDNA libraries were constructed using the identical samples applied for metabolomics analysis and sequenced on the Illumina HiSeq 4000 platform with paired-end and strand-specific libraries. The quality of raw data was controlled and they were trimmed using fastp software with strict parameters: Adapter sequences were completely removed; reads shorter than 50bp were discarded; reads containing more than 5 ambiguous N bases were filtered out; low-quality bases with a Q value below 20 were eliminated; and a 4bp sliding window was adopted for quality trimming, and sequences were trimmed when the average quality in the window was lower than Q20 (Chen et al., 2018). After quality filtering, high-quality clean reads were obtained and aligned to the maize reference genome Zm-B73-REFERENCE-NAM-5.0 using HISAT2 with optimized default parameters. After genome alignment, featureCounts was used to quantify the raw read counts for each gene (Dobin et al., 2013). The raw count matrix was imported into the DESeq2 R package for differential expression analysis. Notably, DESeq2 analysis was performed strictly based on raw integer counts; and FPKM values calculated by StringTie were only used for expression visualization and heatmap plotting, and were not applied for statistical differential analysis. Genes with |log_2_(fold change)|>1.0 and FDR<0.05 were defined as differentially expressed genes (DEGs) (Pertea et al., 2015). The clusterProfiler package was used to conduct GO functional enrichment and KEGG pathway enrichment analyses of DEGs (A et al., 2004; M and S, 2000).

### 2.5 WGCNA analysis and gene network visualization

The weighted gene co-expression network analysis (WGCNA) (Peter and Steve, 2008) package in R was used to construct a weighted gene co-expression network and identify co-expressed gene modules. To ensure a scale-free network, the soft threshold power (β) was determined using the pickSoftThreshold function, with the screening criterion that the scale-free topology fit index R2>0.8 and the mean connectivity remained at a reasonable level. Based on the scale-free topology diagnostic plots, β=18 was selected as the optimal soft threshold for subsequent network construction. Subsequently, the adjacency matrix was calculated and transformed into a topological overlap matrix (TOM) to quantify the co-expression correlation between genes. The topological dissimilarity matrix (dissTOM=1−TOM) was then generated, and hierarchical clustering was performed using the hclust function with the average linkage method. The dynamic tree-cutting algorithm was applied to divide the gene modules. The following key parameters were set according to the WGCNA official tutorial and published studies in maize: deepSplit=2 (moderate sensitivity for module division), minModuleSize=30 (minimum number of genes per module to ensure biological significance of module), and mergeCutHeight=0.25 (threshold for merging similar modules to avoid over-segmentation).

### 2.6 Construction and analysis of the co-expression network of sugar metabolism

Based on the module partitioning results, the Pearson correlation coefficients between each module eigengene (ME) and sugar metabolism-related physiological traits (including the contents of Ara, Gal, Glc, Xyl, and Glc-UA) were calculated using the WGCNA package. Modules with significant correlations (p<0.01) were screened for subsequent analysis, and the weight relationships and connectivity information between genes in each module were extracted. For hub gene screening, two rigorous thresholds were set based on previous WGCNA studies in plants: |KME (Module Membership)|>0.8 (to ensure that genes were highly correlated with the module eigengene, representing core genes of the module) and edge weight>0.2 (to retain genes with strong co-expression correlations, ensuring the reliability of the regulatory network). Meanwhile, modules containing genes annotated to sugar metabolism pathways (e.g., sucrose, starch, and cell wall polysaccharide metabolism) were identified as key modules for sugar metabolism. The protein sequences of genes in key modules were submitted to the PlantTFDB v5.0 database (http://planttfdb.gao-lab.org/) to predict and classify transcription factors (TFs) in each module. Finally, the co-expression network of sugar metabolism was visualized using Cytoscape 3.9.1 software (Habiba et al., 2025). Hub genes were marked in the network (Habiba et al., 2025).

### 2.7 Real-time fluorescent quantitative PCR analysis

Total RNA was extracted from maize stalk tissues, and cDNA was synthesized using HiScript^®^ QRT SuperMix for qPCR (+gDNA wiper) kit. Genomic DNA removal was performed at 42 °C for 2 min, followed by reverse transcription at 50 °C for 15min and inactivation at 85 °C for 2min. The resulting cDNA was diluted for subsequent qPCR analysis. Gene-specific primers were designed using Primer 5 software and synthesized commercially. The *ZmAct2-qRT* gene was used as the internal reference gene. qRT-PCR was performed using ChamQ SYBR qPCR Master Mix on a QuantStudio™ 5 Real-Time PCR System. The reaction system was 10μL, and the amplification procedure included pre-denaturation at 95 °C for 30s, followed by 40 cycles of 95 °C for 10s and 60 °C for 30s. Melting curve analysis was performed from 60 °C to 95 °C. Amplification efficiency for all primer pairs was validated, and amplification curves and melting curves met the requirements of qPCR quality control. The relative expression level of each gene was calculated using the 2^-ΔΔCt^ method. Three biological replicates and three technical replicates were set for each sample to ensure the reliability of the results (**Table 2**).

**Table 2:** Primers for qRT-PCR.

Gene-ID	Primer-name	Primer-sequence (5’-3’)
*ZmAct2-qRT*	ZmAct2-qRT-F	TTCAGCAGTATAGATCGTCAGGTCAG
	ZmAct2-QRT-R	GCGACAGCCGACACCCTTG
Zm00001eb041680	Zm00001eb041680-F	GGGCGTCTTAGTGTTCGTT
	Zm00001eb041680-R	GAGCACCTTCCTCACCTTG
Zm00001eb042110	Zm00001eb042110-F	AGCAATCACATCAGTAGCAGA
	Zm00001eb042110-R	GAATGGGTGGTCAAGACAATA
Zm00001eb057540	Zm00001eb057540-F	AACGGGACCAAACTCAACA
	Zm00001eb057540-R	CTGCCACAGGGAAGATGC
Zm00001eb066790	Zm00001eb066790-F	ATGTTATCTGGGAAGAGGCA
	Zm00001eb066790-R	AACTATTGTGCGGTTGGAG
Zm00001eb106240	Zm00001eb106240-F	GGCAAATGTCCCAATCGT
	Zm00001eb106240-R	GCCCTGGTCTTTCTAATCG
Zm00001eb118120	Zm00001eb118120-F	GACAAAATTCTTGAACGCTATG
	Zm00001eb118120-R	CAGTTTCCTGTATTCGTGGC
Zm00001eb143360	Zm00001eb143360-F	GGAGCACTGCCGCACTTA
	Zm00001eb143360-R	GGTCCGACGACAATGGGT
Zm00001eb009090	Zm00001eb009090-F	ACATCTGGATTGGAGGAACC
	Zm00001eb009090-R	TGAAAATGGCTTGAGGGTAG
Zm00001eb015270	Zm00001eb015270-F	CCGATGCCTTTCGTGTAT
	Zm00001eb015270-R	CTGCTTGGCTGGGTCCTT
Zm00001eb028820	Zm00001eb028820-F	TCGGTGGGGAAACAGGTG
	Zm00001eb028820-R	TCAGCGAAGACGAGGACG

### 2.8 Data statistics and analysis

Phenotypic and structural substance data were statistically analyzed using GraphPad Prism 10 software. Two-way ANOVA was performed, and Tukey’s HSD test was used for *post-hoc* multiple comparisons when significant differences were observed (p<0.05) (Xinyu et al., 2023).

## 3 Results and analysis

### 3.1 Variation analysis of structural substances in stems at different densities

**Figure 1:**
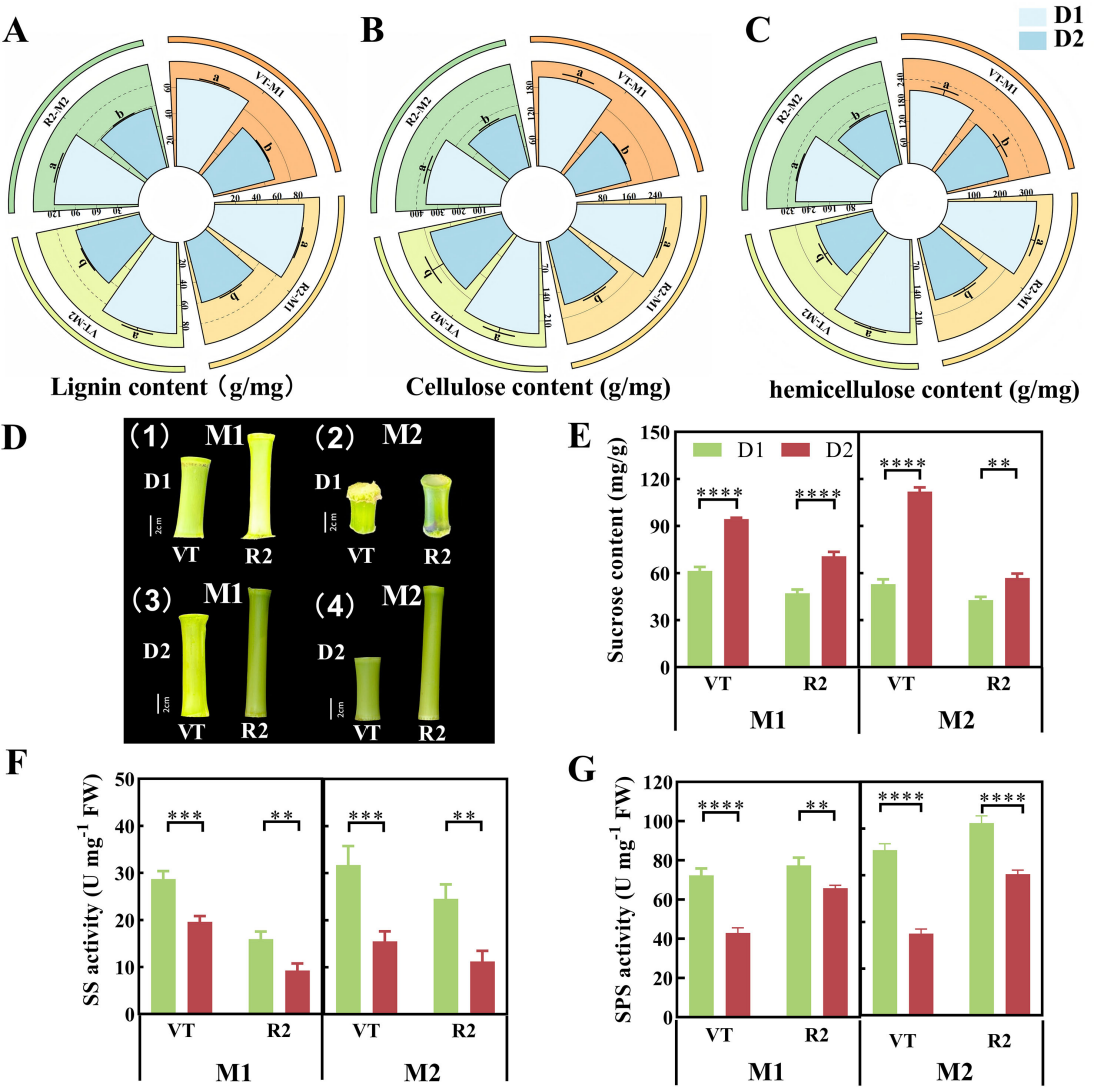
Analysis of cell wall components, sucrose content and sucrose-metabolizing enzyme activities in the third basal internode of maize stalks under different planting densities and developmental stages. **(A)** Lignin content, **(B)** cellulose content, and **(C)** hemicellulose content in the third basal internode. **(D)** Phenotypic characteristics of the third basal internode (Bars = 5 cm). **(E)** Sucrose content. **(F)** Sucrose synthase (SS) activity. **(G)** Sucrose phosphate synthase (SPS) activity.M1 and M2 represent maize inbred lines B12 and J1590, respectively; D1 and D2 represent planting densities of 105,000 and 135,000 plants·ha^-1^, respectively; VT and R2 represent the tasseling stage and grain-filling stage, respectively. Data are presented as the mean ± standard error (SE) with n=6 biological replicates per treatment. Different lowercase letters in panels **(A–C)** indicate significant differences among groups at p < 0.05. In panels **(E–G)**, * indicates p < 0.05, ** indicates p < 0.01, *** indicates p < 0.001, and **** indicates p < 0.0001, as determined by two-way ANOVA followed by Tukey’s multiple comparison test.

Lignin enhances the rigidity of cell walls, cellulose improves stalk flexibility through a microfibril network, and hemicellulose maintains the structural stability of cell walls by linking cellulose and lignin. Together, these three components determine the mechanical strength of stalks. By measuring the content of lignin, cellulose, and hemicellulose in M1 and M2 at different densities, it was found that the three structural components accumulated significantly as the growth stage progressed, with extremely significant differences. Additionally, the content of the three components at density D2 was higher than that at density D1 (**Figures 1A–C**). Please refer to **Equations 1**–**9** for the determination formulas.

### 3.2 Variation analysis of sucrose content and related enzyme activities in stalks at different densities

Through ion chromatography (IC) analysis of the third internode at the base of M1 and M2 (**Figure 1D**) under D1 and D2 treatments during two developmental stages, 5 monosaccharide metabolites were identified. All of these are derived from sucrose hydrolysis. Therefore, sucrose content and sucrose-related enzyme activities in the third internode of the stalk base under different treatments were measured, revealing that sucrose content and related enzyme activities exhibit significant sensitivity to changes across different developmental stages. As planting density increased, sucrose accumulation in the third internode of the stalk base showed an increasing trend, with extremely significant differences (**Figure 1E**). As planting density increased, SS activity decreased gradually, showing extremely significant differences among different developmental stages (**Figure 1F**). With the increase of planting density, SPS activity presented a significant downward trend; meanwhile, SPS activity increased continuously with the advancement of growth stages (**Figure 1G**). Please refer to **Equations 10**–**12** for the determination formulas.

### 3.3 Changes in the content of stalk sugar metabolites at different densities

**Figure 2:**
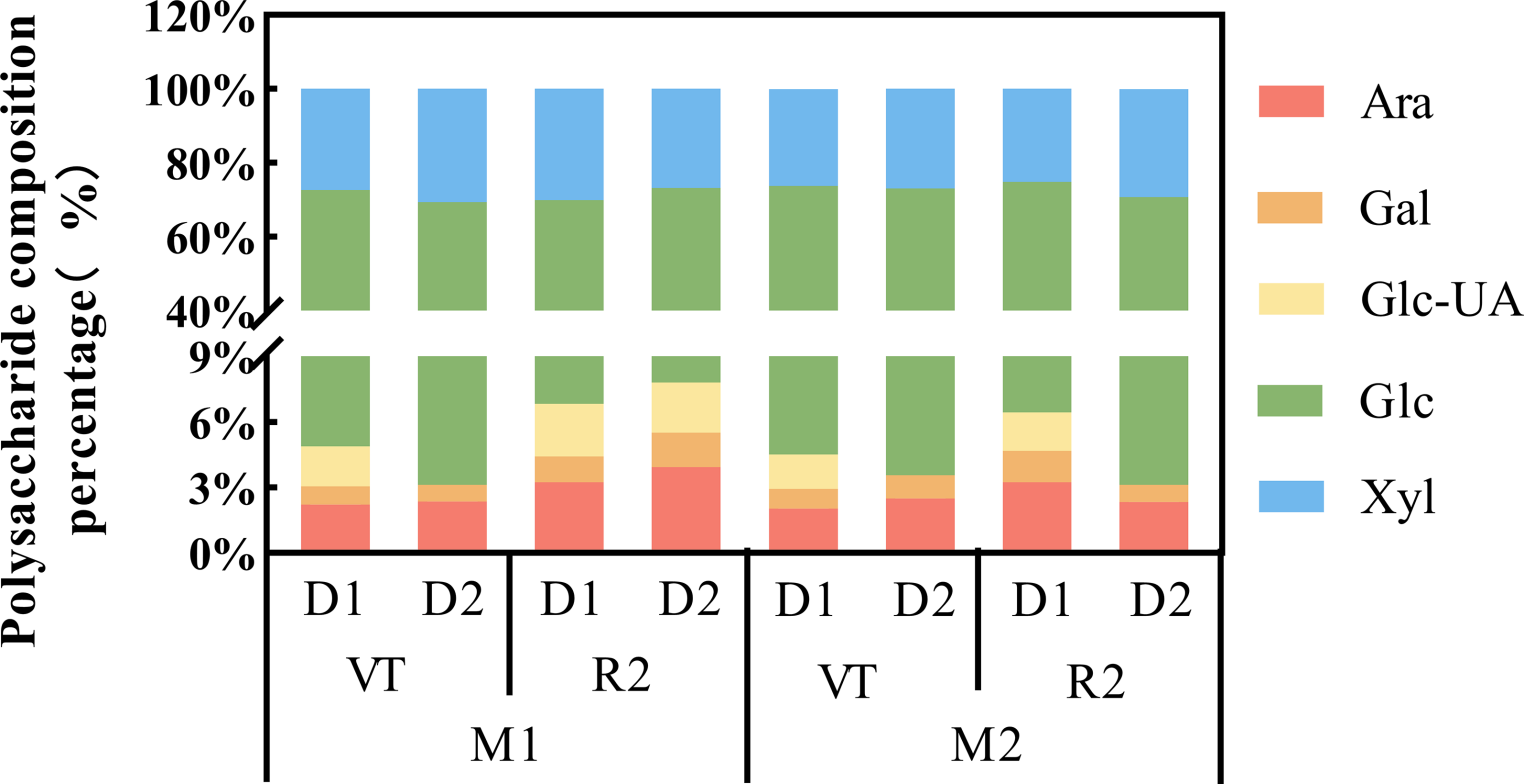
Percentage composition of major polysaccharide components in the third basal internode of maize stalks under different planting densities and developmental stages. The stacked bar chart shows the relative proportions of arabinose (Ara), galactose (Gal), galacturonic acid (Glc-UA), glucose (Glc), and xylose (Xyl). M1 and M2 represent maize inbred lines B12 and J1590, respectively; D1 and D2 represent planting densities of 105,000 and 150,000 plants·ha^-1^, respectively; VT and R2 represent the tasseling stage and grain-filling stage, respectively. The y-axis is broken to clearly display the low-abundance sugar components. Data are presented as the mean of three biological replicates, with error bars representing the standard error of the mean (SE). Statistical significance was determined by two-way ANOVA followed by Tukey’s multiple comparison test (p < 0.05).

Quantitative analysis of 5 sugar metabolites (**Figure 2**) revealed that glucose content was highest in the third internode of the maize base under different treatments during the VT-R2 stage, accounting for 60%–70% of total metabolites. In contrast, galacturonic acid accumulation showed an opposite trend, with the lowest content. Under D1 treatment, neither M1 nor M2 produced galacturonic acid during the VT stage. Please refer to **Equation 13** for the determination formulas.

### 3.4 Analysis of transcriptome sequencing results

To further study the gene expression profiles of the third internode at the base of maize stalks at different densities and developmental stages, 24 cDNA libraries were constructed and transcriptome sequencing was performed. A total of 182.45Gb of raw sequencing data was obtained, with 178.64Gb of high-quality clean reads after quality control, averaging 7.44Gb per sample; the Q20 and Q30 values for each sample ranged from 99.02% to 99.33% and 96.02% to 97.76%, respectively, meeting the standards for gene sequencing samples; 92.96% to 95.78% of the reads were mapped to the maize reference genome, with 88.84% to 91.45% mapped to unique sites and 3.58% to 4.23% mapped to multiple sites. Detailed sequencing quality statistics are provided in **Supplementary Tables S1**, **S2**.

**Figure 3:**
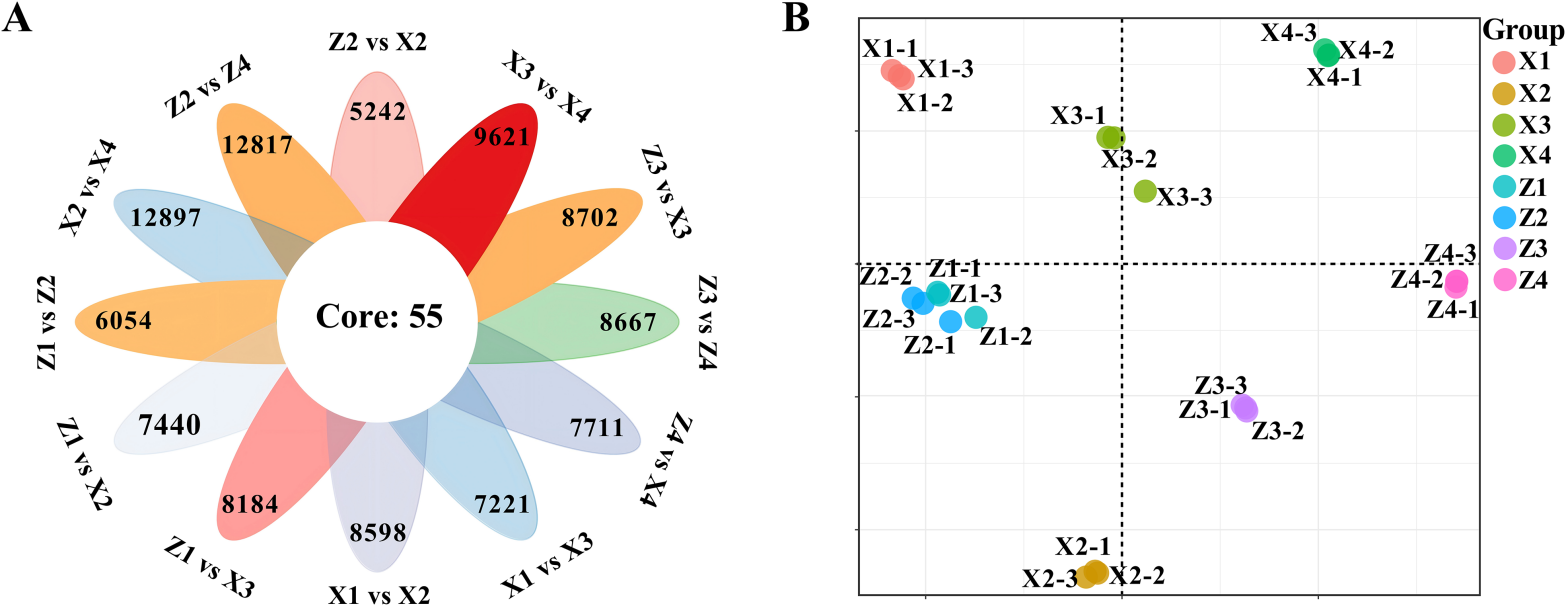
Analysis of differentially expressed genes and sample expression clustering in the third basal internode of maize stalks under different treatments. **(A)** Flower plot showing the number of differentially expressed genes (DEGs) in 12 comparison groups, with 55 core DEGs shared by all comparison groups. DEGs were screened with the threshold of |log_2_(fold change)| ≥ 1 and false discovery rate (FDR) < 0.05. **(B)** Principal component analysis (PCA) plot based on normalized gene expression levels, showing the clustering of 24 samples.M1 and M2 represent maize inbred lines B12 and J1590, respectively; D1 and D2 represent planting densities of 105,000 and 135,000 plants·ha^-1^, respectively; VT and R2 represent the tasseling stage and grain-filling stage, respectively.Z1/Z2, Z3/Z4, X1/X2, and X3/X4 represent M1 at VT stage, M1 at R2 stage, M2 at VT stage, and M2 at R2 stage under D1/D2 densities, respectively. Each group contains 3 biological replicates, and the sample IDs in panel **(B)** correspond to the treatment groups as detailed above.

By comparing the differences between samples subjected to different treatments, a total of 39,757 differentially expressed genes were identified. The number of differentially expressed genes varied from 5,242 to 12,897 across different comparison groups. Analysis of different comparison groups revealed that 55 differentially expressed genes were common across all 12 groups (**Figure 3A**). Principal component analysis was used to reduce the dimensionality of gene expression data from different comparison groups (**Figure 3B**). The samples from different comparison groups exhibited a certain clustering trend. Samples in the same comparison group showed high similarity in gene expression, while there were significant differences between different samples.

### 3.5 Statistics on the number of differentially expressed genes

**Figure 4:**
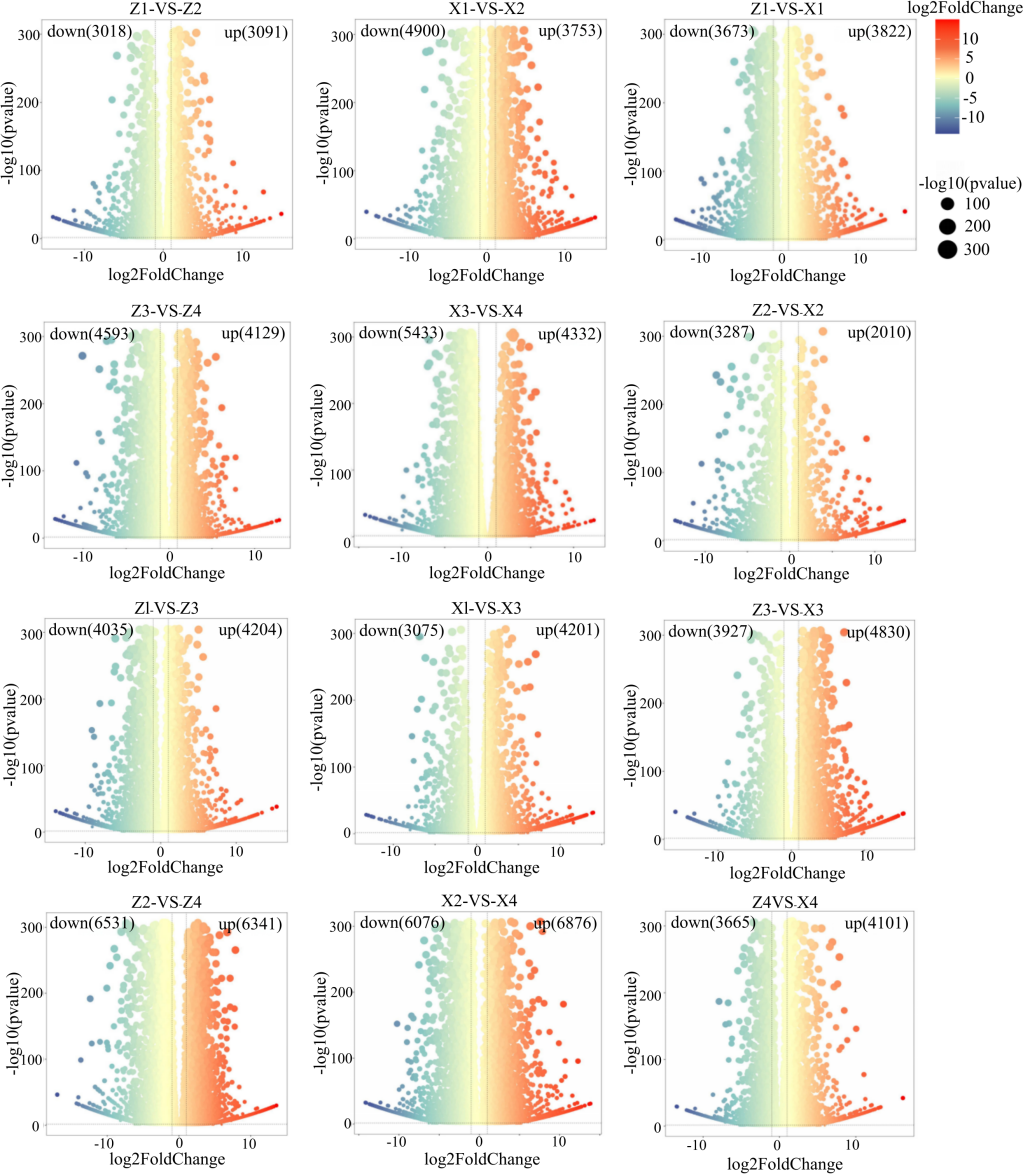
Volcano plots of differentially expressed genes (DEGs) in the third basal internode of maize stalks under 12 different comparison groups. The x-axis represents the log_2_-transformed fold change (log_2_FC) of gene expression, and the y-axis represents the -log_10_-transformed p-value [-log_10_ (pvalue)]. DEGs were screened with the threshold of |log_2_FC| ≥ 1 and false discovery rate (FDR) < 0.05, with up-regulated genes shown in red and down-regulated genes shown in blue. The color of the dots indicates the log_2_FC value, and the size of the dots indicates the -log_10_(pvalue) value.M1 and M2 represent maize inbred lines B12 and J1590, respectively; D1 and D2 represent planting densities of 105,000 and 135,000 plants·ha^-1^, respectively; VT and R2 represent the tasseling stage and grain-filling stage, respectively.Z1/Z2, Z3/Z4, X1/X2, and X3/X4 represent M1 at VT stage, M1 at R2 stage, M2 at VT stage, and M2 at R2 stage under D1/D2 densities, respectively. Each comparison group contains 3 biological replicates.

There are differences in the expression patterns of genes between the two varieties (**Figure 4**). In the four comparison groups Z1-vs-X1, Z2-vs-X2, Z3-vs-X3, and Z4-vs-X4, there were 12,954, 5,297, 8,757, and 7,766 differentially expressed genes, respectively. Among them, there were 6,076, 3,287, 3,927, and 3,665 downregulated genes, and 6,876, 2,101, 4,830, and 4,101 upregulated genes, respectively.

Comparing the differentially expressed genes of M1, the number of differentially expressed genes in Z1-vs-Z2, Z3-vs-Z4, Z1-vs-Z3, and Z2-vs-Z4 was 6,109, 8,722, 8,239, and 12,872, respectively; among them, the number of downregulated genes was 3,018, 4,593, 4,035, and 6,531, respectively; and the number of upregulated genes was 3,091, 4,129, 4,204, and 6,341, respectively. In M1, as planting density increased during the same growth stage, the number of differentially expressed genes also increased, indicating that maize stalks respond to density through multiple genes, alleviating density-induced stress through their own gene expression.

Comparing the differentially expressed genes of M2, the number of differentially expressed genes for X1-vs-X2, X3-vs-X4, X1-vs-X3, and X2-vs-X4 was 8,653, 9,676, 7,276, and 12,952, respectively; among them, the number of downregulated genes was 4,900, 5,344, 3,075, and 6,076, respectively; and the number of upregulated genes was 3,753, 4,332, 4,201, and 6,876, respectively. In M2, as planting density increased during the same growth stage, the number of differentially expressed genes increased; however, at density D1, as the growth stage progressed, the number of upregulated and downregulated genes in M2 decreased.

### 3.6 Functional enrichment analysis of differentially expressed genes

**Figure 5:**
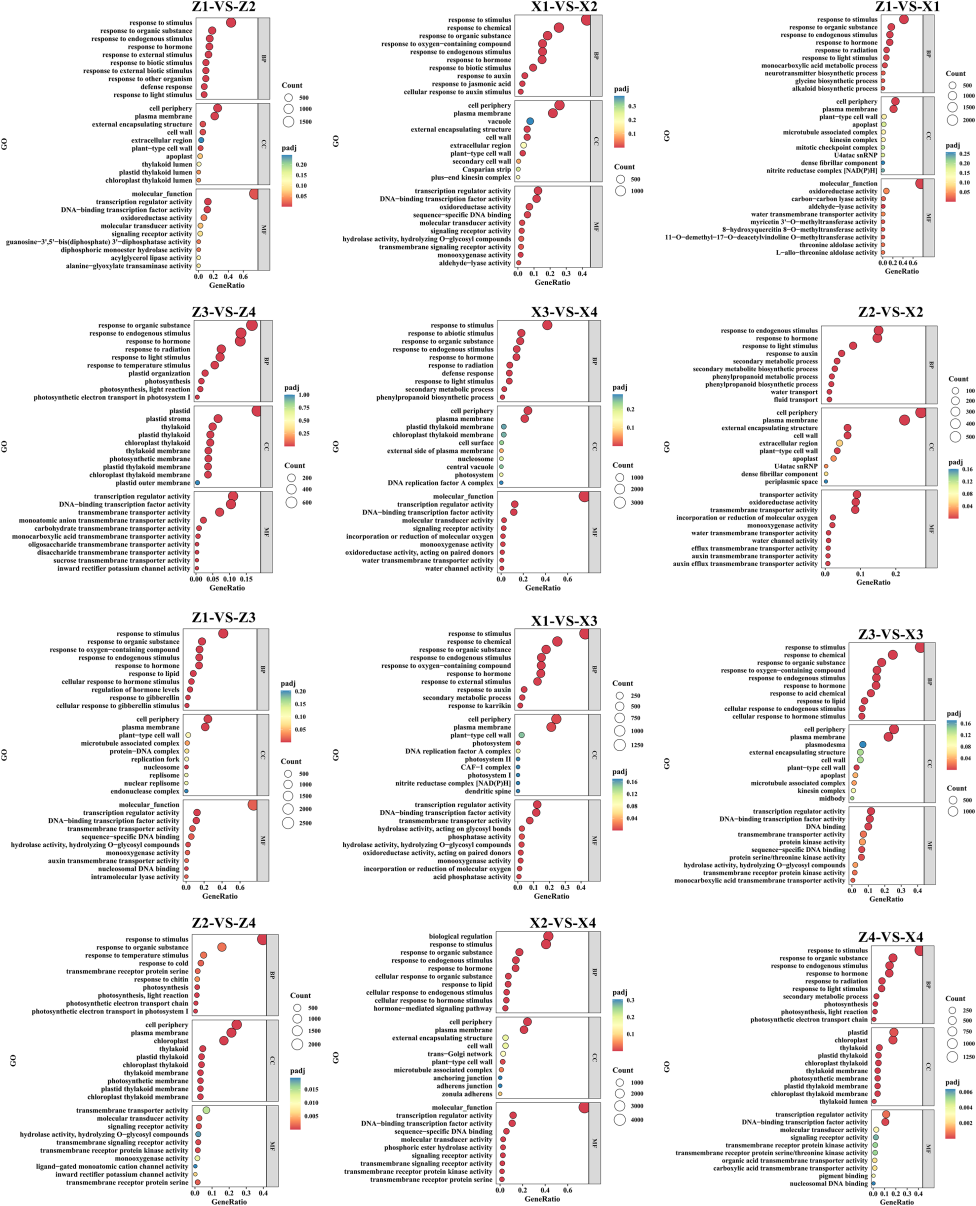
GO functional enrichment analysis of differentially expressed genes (DEGs) in 12 comparison groups. The dot plots show the top significantly enriched Gene Ontology (GO) terms, including biological process (BP), cellular component (CC), and molecular function (MF). The x-axis represents the gene ratio (the proportion of DEGs annotated to the term), and the y-axis represents the GO term. The color of the dots indicates the adjusted p-value (padj), and the size of the dots indicates the number of DEGs (Count) enriched in the term. Enrichment analysis was performed with the threshold of padj < 0.05 using the clusterProfiler package in R.M1 and M2 represent maize inbred lines B12 and J1590, respectively; D1 and D2 represent planting densities of 105,000 and 135,000 plants·ha^-1^, respectively; VT and R2 represent the tasseling stage and grain-filling stage, respectively.Z1/Z2, Z3/Z4, X1/X2, and X3/X4 represent M1 at VT stage, M1 at R2 stage, M2 at VT stage, and M2 at R2 stage under D1/D2 densities, respectively. Each panel corresponds to the comparison group labeled at the top.

To understand the main biological functions and regulatory patterns of the screened differentially expressed genes, GO and KEGG enrichment analyses were performed. The GO enrichment results showed that the main categories of biological processes included responses to stimuli, cellular responses to hormonal stimuli, secondary metabolic processes, and photosynthesis; categories such as external envelope structures and plastid and chloroplast-related structures were enriched in cellular components; and the highest enrichment levels were observed in molecular functions such as DNA-binding transcription factor activity, regulatory activity of transcription factor, and transmembrane transporter activity (**Figure 5**).

**Figure 6:**
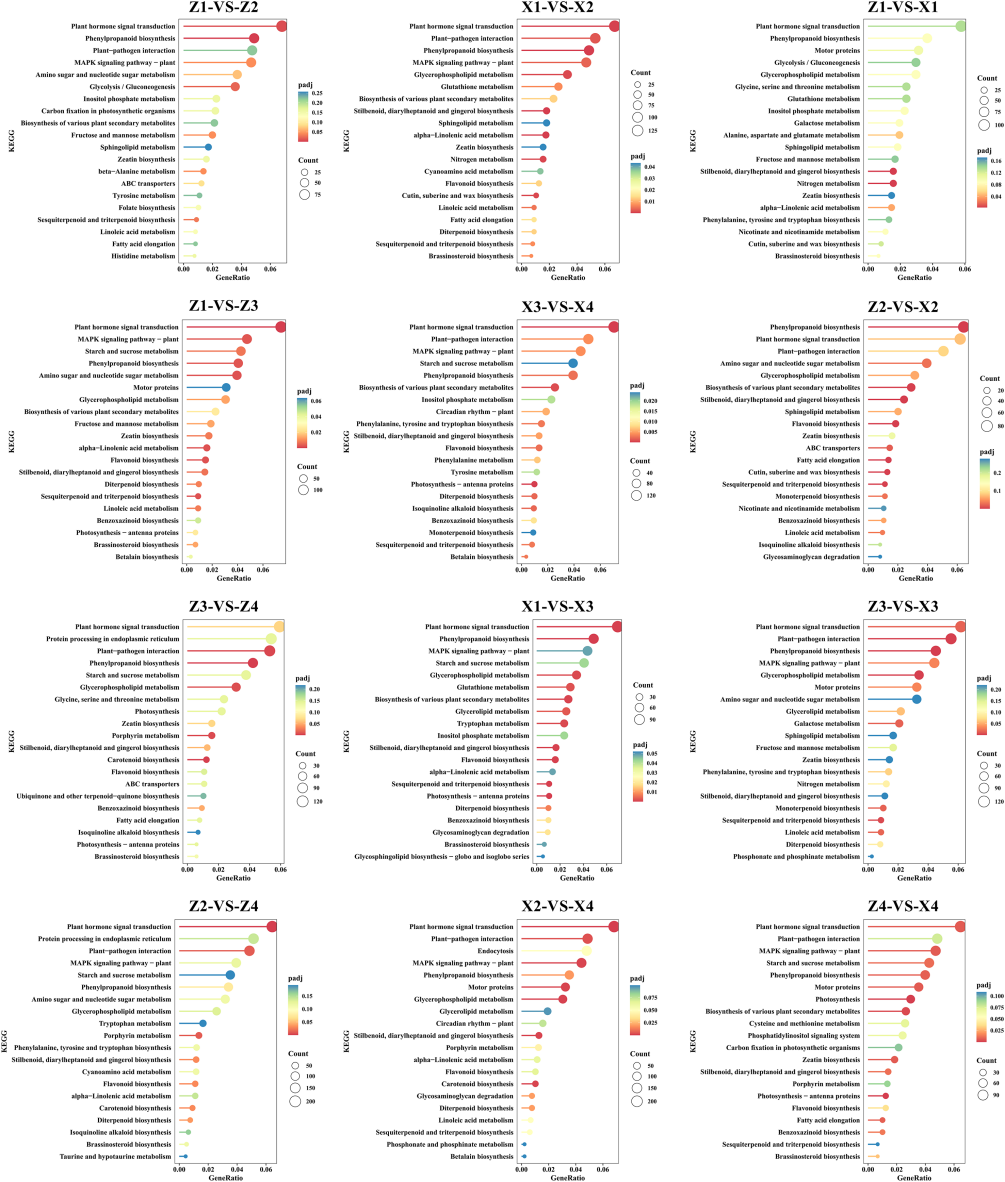
KEGG pathway enrichment analysis of differentially expressed genes (DEGs) in 12 comparison groups. The dot plots show the top significantly enriched Kyoto Encyclopedia of Genes and Genomes (KEGG) pathways. The x-axis represents the gene ratio (the proportion of DEGs annotated to the pathway), and the y-axis represents the KEGG pathway term. The color of the dots indicates the adjusted p-value (padj), and the size of the dots indicates the number of DEGs (Count) enriched in the pathway. Enrichment analysis was performed with the threshold of padj < 0.05 using the clusterProfiler package in R.M1 and M2 represent maize inbred lines B12 and J1590, respectively; D1 and D2 represent planting densities of 105,000 and 135,000 plants·ha^-1^, respectively; VT and R2 represent the tasseling stage and grain-filling stage, respectively.Z1/Z2, Z3/Z4, X1/X2, and X3/X4 represent M1 at VT stage, M1 at R2 stage, M2 at VT stage, and M2 at R2 stage under D1/D2 densities, respectively. Each panel corresponds to the comparison group labeled at the top.

KEGG enrichment analysis of the top 20 entries revealed that “Plant hormone signal transduction” was significantly enriched in all KEGG enrichments, indicating that it is a core pathway and plays a crucial role across different planting densities and growth stages. Enrichment terms related to carbohydrate metabolism, such as “Starch and sucrose metabolism,” “Glycolysis/Gluconeogenesis,” “Fructose and mannose metabolism,” “Inositol phosphate metabolism,” and “Amino sugar and nucleotide sugar metabolism,” are also significantly enriched (**Figure 6**).

### 3.7 Weighted gene co-expression network analysis

**Figure 7:**
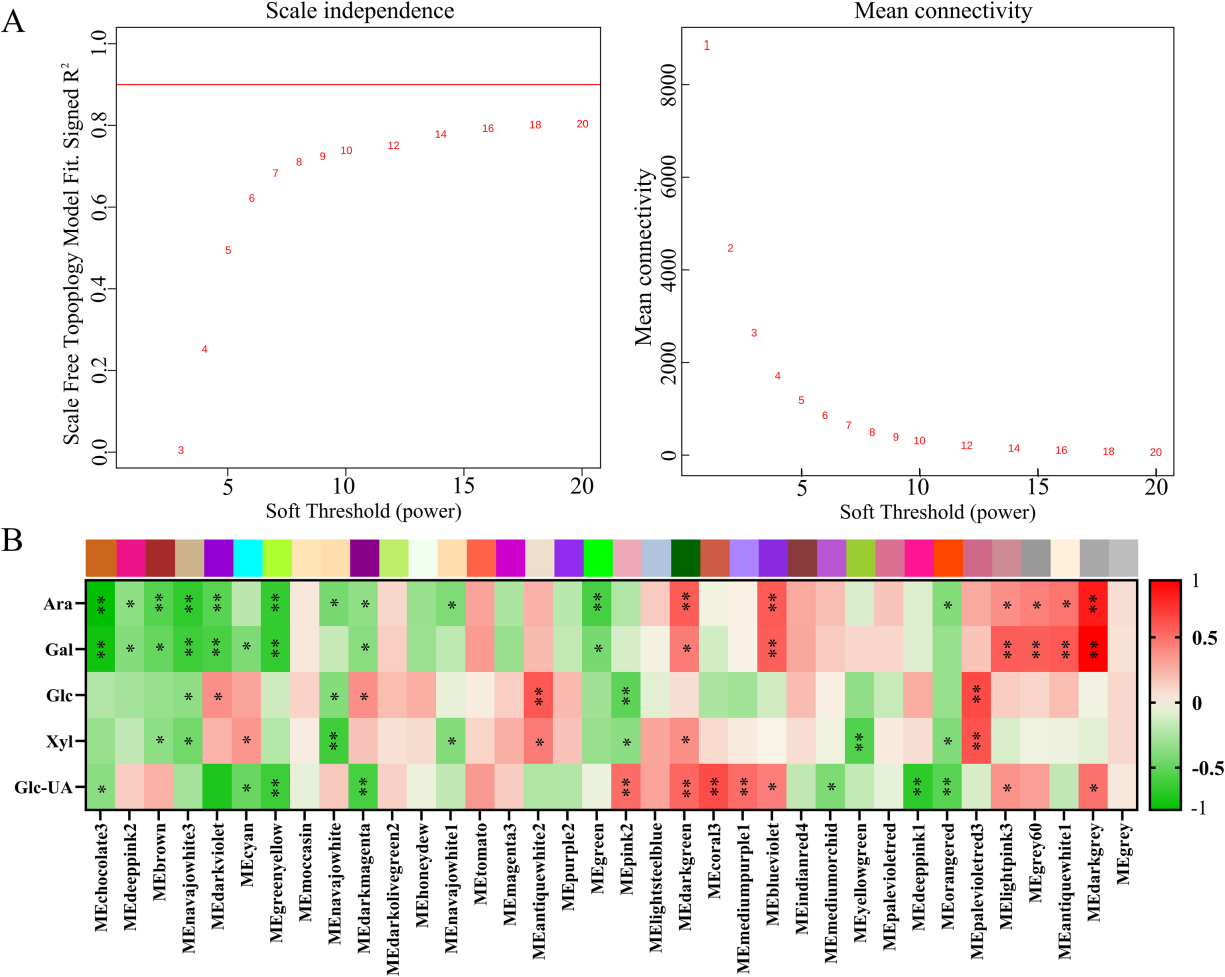
Weighted gene co-expression network analysis (WGCNA) of differentially expressed genes (DEGs) associated with monosaccharide metabolites. **(A)** Selection of the optimal soft-threshold power (β) for WGCNA network construction. The left panel shows the scale-free topology fit index (R^2^) for different β values, with the red line indicating the threshold of R^2^ > 0.8. The right panel shows the mean connectivity of genes under different β values. The optimal β was determined as 18 to ensure the network conforms to the scale-free topology. **(B)** Module-trait relationship (MTR) heatmap showing the Pearson correlation between module eigengenes (MEs) and monosaccharide contents. The color of each cell represents the correlation coefficient (red: positive correlation, green: negative correlation), and the number in the cell represents the corresponding p-value. * indicates p < 0.05, ** indicates p < 0.01. Ara, Gal, Glc-UA, Glc, and Xyl represent arabinose, galactose, galacturonic acid, glucose, and xylose, respectively.

To construct a reliable weighted gene co-expression network, the optimal soft threshold power (β) was determined using the scale-free topology criterion. As shown in **Figure 7A**, the scale-free topology model fit index (R2) gradually increased with the increase of soft threshold power. When β=18, the R2 reached 0.85, which met the recommended threshold (R2>0.8) for scale-free network construction, and the corresponding mean connectivity remained at a reasonable level (mean connectivity≈2.3), ensuring that the network had sufficient biological connectivity. Therefore, β=18 was selected as the optimal soft threshold for subsequent analysis. Using the selected soft threshold, WGCNA was performed on 25,022 high-confidence genes with FPKM>1 in at least one sample, and a total of 36 distinct co-expressed gene modules were obtained (**Figure 7B**). The size of the modules varied greatly: MEchocolate3, the largest module, contained 8,154 genes, while MEgrey (a set of genes that could not be assigned to any module), the smallest module, contained only 17 genes. Subsequently, the correlation between module eigengenes and sugar metabolism-related physiological traits was analyzed. Multiple modules showed significant correlations with key sugar components: For example, the MEchocolate3 and ME green yellow modules were significantly positively correlated with arabinose (Ara) content (r>0.6, p<0.01), while the ME blue violet module was significantly negatively correlated with Ara content (r<−0.6, p<0.01). Similar significant associations were also observed between specific modules and other sugar components, including galactose (Gal), glucose (Glc), xylose (Xyl), and glucuronic acid (Glc-UA), indicating that these modules may play key regulatory roles in sugar metabolism in maize stalks.

### 3.8 Analysis of key differentially expressed genes in the sugar metabolism pathway

**Figure 8:**
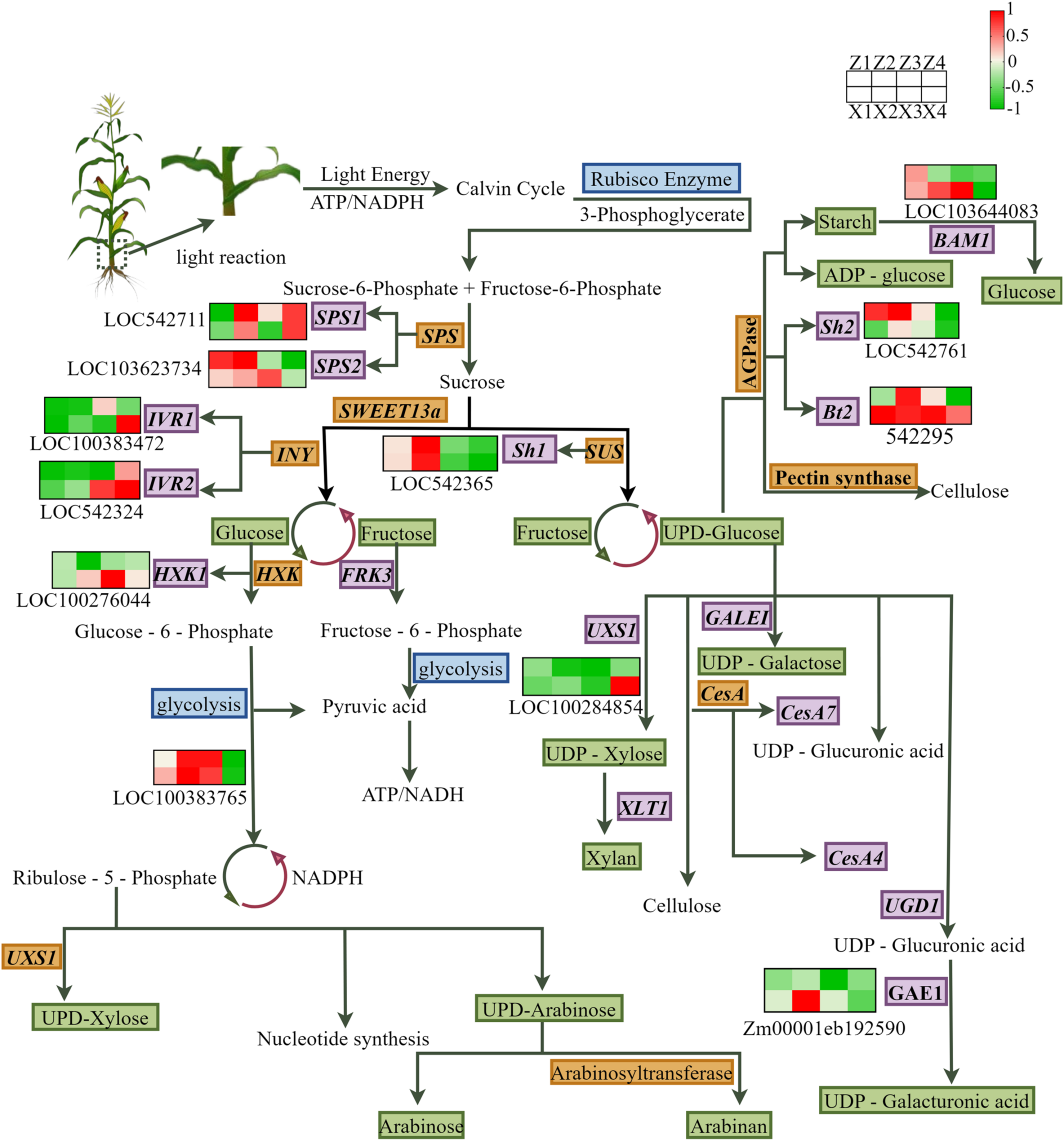
Schematic diagram of sugar metabolism and cell wall biosynthesis pathway in the basal internodes of maize, with expression heatmaps of key differentially expressed genes (DEGs).The pathway shows the core metabolic processes from photosynthesis and sucrose metabolism to cell wall polysaccharide (cellulose, hemicellulose, pectin) synthesis. Arrows indicate the direction of metabolic flow. The heatmap next to each gene shows the normalized expression level of the gene in 8 treatment groups: Z1/Z2 (M1, VT stage, D1/D2 densities), Z3/Z4 (M1, R2 stage, D1/D2 densities), X1/X2 (M2, VT stage, D1/D2 densities), X3/X4 (M2, R2 stage, D1/D2 densities). The color scale represents the expression level, with red indicating high expression and green indicating low expression.M1 and M2 represent maize inbred lines B12 and J1590, respectively; D1 and D2 represent planting densities of 105,000 and 135,000 plants·ha^-1^, respectively; VT and R2 represent the tasseling stage and grain-filling stage, respectively. DEGs were screened with the threshold of |log_2_FC| ≥ 1 and FDR < 0.05.

At different densities in the third internode of the maize stalk base, a total of 12 differentially expressed genes related to sugar metabolism were identified across two growth and development stages (**Figure 8**). As the first key enzyme in the sugar metabolism mechanism, SPS is the rate-limiting enzyme for sucrose synthesis and directly influences sucrose accumulation in the stalk. The expression level of the *SPS1* was elevated with advancing growth stages at density D2, while *SPS2* exhibited the opposite expression pattern; *IVR1* and *IVR2* showed little change in expression levels at different densities as the growth stage progressed in M1, but expression levels were elevated as the growth stage progressed at density D2 in M2; and *BAM1* and *Sh2* showed a decreasing trend in expression levels as the growth stage progressed and the stalk gradually aged. *GAE1* showed no significant change in expression levels in M1 as the growth stage progressed, but expression levels showed an upward trend at density D1 in M2.

### 3.9 Construction and analysis of the regulatory network of key genes involved in sugar metabolism biosynthesis

**Figure 9:**
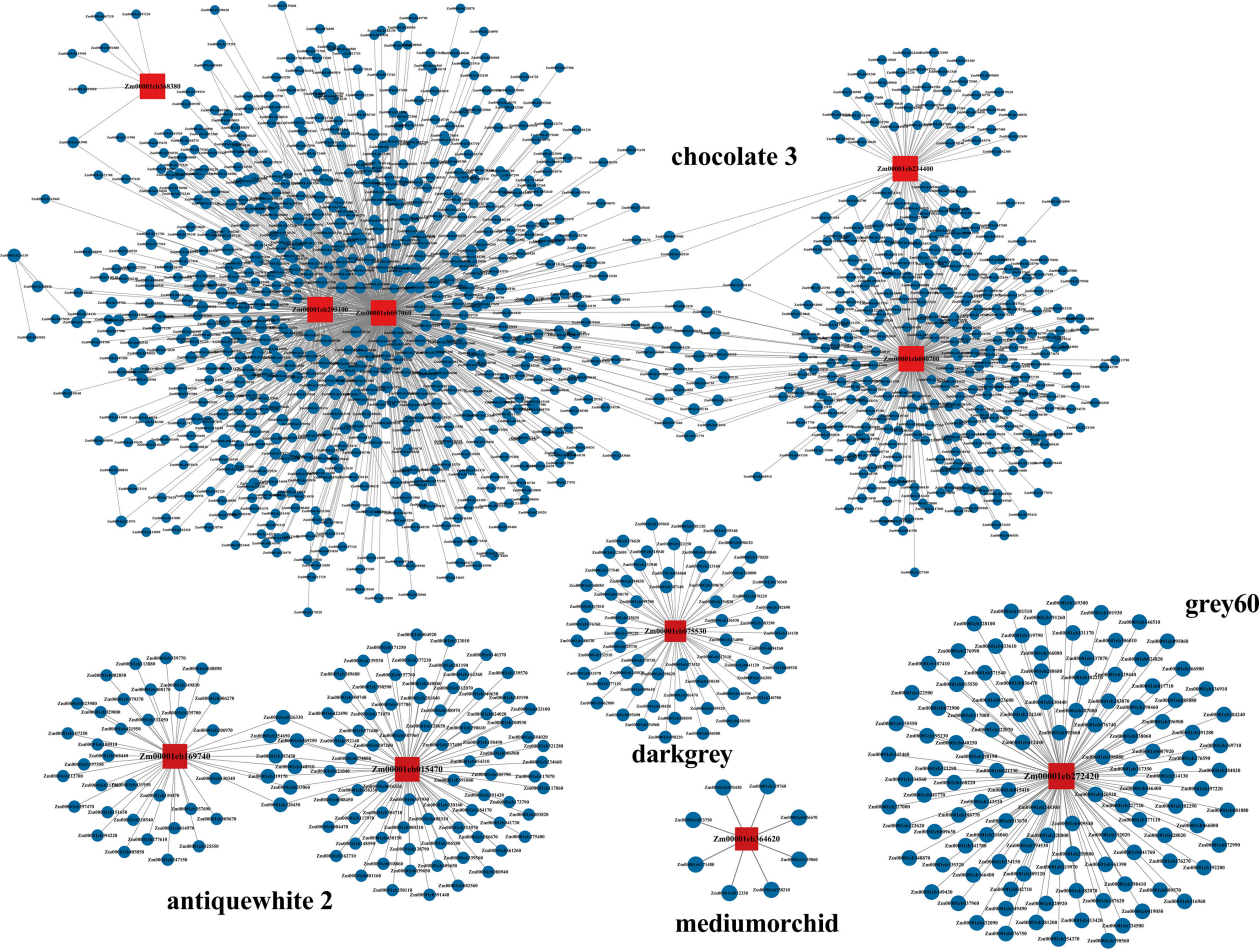
Co-expression regulatory networks of sugar metabolism-related genes and transcription factors (TFs) in the basal internodes of maize, constructed from 5 key WGCNA modules (chocolate3, antiquewhite2, dark grey, grey60, medium orchid). Red squares represent hub differentially expressed genes (DEGs) related to sugar metabolism in each module, and blue circles represent differentially expressed TFs that have co-expression interactions with sugar metabolism pathway genes. Lines between nodes indicate significant co-expression relationships, screened with the thresholds of |KME| > 0.8 and weight > 0.2. The networks were constructed based on WGCNA analysis and visualized using Cytoscape software.

The analysis revealed that there were 5, 2, 1, 1, and 1 sugar metabolism-related differentially expressed genes in the chocolate3, antiquewhite2, dark grey, grey60, and medium orchid modules, respectively. To identify regulatory genes involved in sugar metabolism in the third internode of the maize stalk base, transcription factors with KME>|0.8| and weight>0.2 relative to the aforementioned 12 differentially expressed genes were screened from the five modules (Two genes did not yield transcription factors meeting the criteria). Using the 10 differentially expressed genes as core genes, five interaction networks were constructed using Cytoscape software (**Figure 9**). The detailed information of these 10 core genes, including gene ID, gene symbol, transcription factor family, and corresponding protein ID, is summarized in **Supplementary Table S3**. Among the five modules, the number of differentially expressed transcription factors with interaction relationships with differentially expressed genes related to sugar metabolism were 1,312, 127, 113, 61, and 8, respectively.

### 3.10 qRT-PCR validation of the expression patterns of differentially expressed genes in transcriptomic sequencing

**Figure 10:**
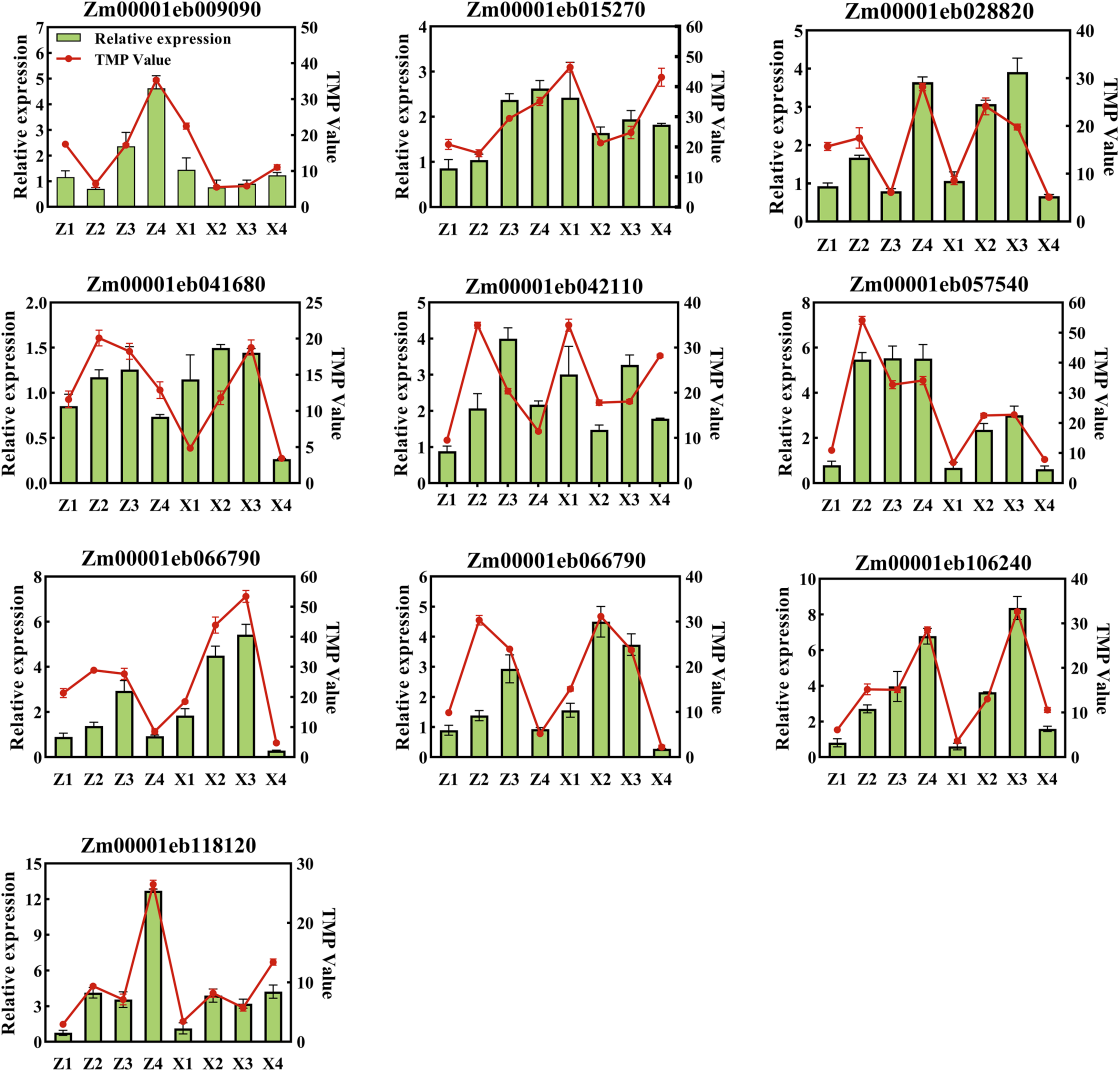
Validation of RNA-seq data by qRT-PCR for 10 candidate genes in the third basal internode of maize stalks. The green bars represent the relative expression levels of genes detected by qRT-PCR (left y-axis), and the red lines represent the transcript abundance (TMP values) from RNA-seq (right y-axis). Error bars represent the standard error of the mean (SE) of three biological replicates.M1 and M2 represent maize inbred lines B12 and J1590, respectively; D1 and D2 represent planting densities of 105,000 and 135,000 plants·ha^-1^, respectively; VT and R2 represent the tasseling stage and grain-filling stage, respectively.Z1/Z2, Z3/Z4, X1/X2, and X3/X4 represent M1 at VT stage, M1 at R2 stage, M2 at VT stage, and M2 at R2 stage under D1/D2 densities, respectively. The qRT-PCR was performed using the 2^-^ΔΔCT method with Actin as the internal reference gene, and the results showed a high correlation with RNA-seq data, confirming the reliability of the transcriptome sequencing results.

Twelve differentially expressed genes (DEGs) that were either upregulated or downregulated in two varieties at different densities and different growth stages were selected for qRT-PCR analysis to validate the accuracy of the RNA-seq data (**Figure 10**). Comparing qRT-PCR results with sequencing results, the relative expression levels of 10 genes from the two varieties at different densities and growth stages were consistent with transcriptomics sequencing data, confirming the accuracy of RNA-seq data. Therefore, the selected genes can serve as candidate genes for further investigation into the sugar metabolism mechanisms of maize stalks at different densities.

## 4 Discussion

### 4.1 Response of structural substances and sucrose content in maize stalks to planting density

The basal internodes of maize stalks serve as critical organs for supporting the plant and temporarily storing photosynthetic products. The dynamic changes in sucrose content and related enzyme activities in these internodes are of significant importance for the plant’s response to planting density stress (Schnyder, 1993). Experimental findings indicate that sucrose content in the third basal internode significantly increases as planting density increases from D1 to D2 and maintains this trend throughout the developmental process from VT to R2. This result is highly consistent with the theoretical framework of the restructuring of plant source-sink relationships under high-density conditions (Koffi et al., 2022). Similarly, Cui et al (Jingjing et al., 2022). observed in their study on maize tolerance to high density that sucrose accumulation in the basal internodes of maize significantly increased under high-density conditions, and this accumulation was closely related to the mechanical strength of stalk, potentially enhancing stalk resistance to lodging through the promotion of cell wall component synthesis. It is worth noting that lignin, cellulose, and hemicellulose play a crucial role in the mechanical strength of stalk as the primary components of stalk cell walls. Lignin enhances stalk rigidity, cellulose improves stalk toughness by forming a microfibril network, and hemicellulose plays a crucial role in linking cellulose and lignin. Together, these three components maintain the structural stability of stalk, thus influencing the plant’s resistance to lodging (Carpita and Gibeaut, 1993).

From an enzyme mechanism perspective, changes in SS and SPS activity are central to the regulation of sucrose accumulation. In the experiments, SS activity decreased significantly with increasing density and exhibited extremely significant differences in both growth stages. As the key enzyme catalyzing the breakdown of sucrose into UDP-glucose and fructose, the decrease in SS activity directly inhibits sucrose degradation, which may be one of the important reasons for sucrose accumulation under high density conditions (Hong-Ni et al., 2010; Hong et al., 2022). Notably, SPS activity exhibited genotype-specific responses. SPS activity in B12 significantly decreased with increasing density, while the opposite trend was observed in J1590. This suggests that J1590 may maintain a higher sucrose synthesis rate under high density conditions by enhancing SPS activity, while B12 relies more on inhibiting degradation pathways to achieve sucrose accumulation. From VT to R2, as grains begin to fill, the plant’s demand for carbon increases sharply, theoretically promoting the redistribution of sucrose from stalks to grains (Peter, 2003; Yang et al., 2022). However, in the experiment, it was found that the sucrose content in stalks at high density remained significantly higher than that at low density even at the R2 stage, and SS activity continued to remain at a low level. This suggests that high density may delay the remobilization of sucrose in stems by inhibiting the activation of sucrose degradation enzymes.

### 4.2 Expression of sugar metabolites and key genes in maize stalks at different densities

Quantitative analysis of sugar metabolites indicates that glucose constitutes the highest proportion of sugar metabolites in the basal internodes of maize during the VT-R2 stage, accounting for 60–70% of total metabolites and dominating the profile, while galacturonic acid constitutes the lowest proportion. This aligns with the distribution patterns of different sugars in plant metabolism reported in previous studies. The differences in the content of various sugar compounds reflect their distinct roles and metabolic flux variations during maize growth and development (Couture et al., 2021). In KEGG enrichment analysis, plant hormone signal transduction emerged as the core pathway, spanning all comparison groups, potentially participating in sugar metabolism and growth and development by regulating gene expression. Additionally, pathways related to sugar metabolism, such as starch and sucrose metabolism and glycolysis, were significantly enriched, confirming that sugar metabolism is a complex process regulated by multiple pathways in a synergistic manner, serving as the core mechanism in response to density changes.

In this study, nine differentially expressed genes associated with sugar metabolism in the third basal internodes of maize stalks were clearly identified. The expression patterns of these genes were significantly influenced by planting density and growth stage, and showed marked differences among different genotypes. Among them, as a key rate-limiting enzyme in sugar metabolism, SPS directly affects sucrose accumulation in stalks (Roitsch et al., 2000; Roopendra et al., 2018; Datir and Joshi, 2016). Experiments revealed that *SPS1* exhibited upregulation in expression levels as the growth stage progressed under D2 density conditions, while *SPS2* exhibited the opposite expression pattern. These differences in gene expression may be closely related to the adaptability of different varieties to planting density and the physiological requirements of the growth stage. The expression levels of *IVR1* and *IVR2* in M1 did not show significant changes with the progression of the growth stage at different densities, but M2 showed an elevation in expression levels with the progression of the growth stage at D2. Invertase catalyzes the hydrolysis of sucrose into glucose and fructose, playing an indispensable role in plant carbohydrate metabolism (Wu et al., 2025). The differences in its expression patterns may reflect distinct strategies employed by different varieties in regulating carbohydrate metabolism in response to changes in planting density. In some plants, enhanced invertase activity facilitates increased absorption and utilization of sugars by storage organs (Xiangping et al., 2021; Bachar et al., 2022). In maize stalks, changes in *IVR* expression may influence the hydrolysis rate of sucrose, thus affecting the metabolic flow of sugars in the stalks. This has significant implications for the regulation of sugar metabolism in maize stalks under different environmental conditions.

The expression levels of the *BAM1* and *Sh2* genes show a downward trend as the biological stage progresses. *BAM1* is involved in the hydrolysis of starch, and a reduction in its activity may lead to decreased starch hydrolysis, thus affecting sugar production (Monroe et al., 2014; Monroe, 2020). The protein encoded by *Sh2* also plays a key role in the metabolic pathway of sucrose (Giroux and Hannah, 1994). The downregulation of these two genes may indicate a decrease in the activity of starch and sucrose metabolism in the stalks during the later stages of maize development. In wheat (Du et al., 2020), it has also been observed that the expression levels of key genes involved in starch and sucrose metabolism become lower as plants age, leading to weakened sugar metabolic activity. The expression pattern of the *ZmGAE1* gene also deserves attention. *ZmGAE1* is involved in the synthesis of UDP-glucuronic acid, and changes in its expression may affect the glucuronic acid pathway, thus influencing the synthesis of cell wall components (Lerouxel et al., 2006). At different densities and growth stages, the expression of *ZmGAE1* may undergo corresponding changes, which may represent a regulatory mechanism for maize stems to adapt to changes in planting density. For example, under high-density conditions, to maintain structural stability of the stem and enhance resistance to lodging, *ZmGAE*1 may regulate UDP-glucuronic acid synthesis, participate in the synthesis of cell wall components such as pectin, and thereby influence the mechanical strength of the stem (Lerouxel et al., 2006). However, further in-depth research is required to clarify the specific functions of *ZmGAE1* in maize sugar metabolism and stalk development, as well as its precise role in the planting density response mechanism.

### 4.3 Construction of a regulatory network for maize stalk sugar metabolism pathways to help elucidate density response mechanisms

By constructing a regulatory network of key genes involved in sugar metabolism, we identified interactions between transcription factors and sugar metabolism-related genes across multiple modules. These interactions are crucial for understanding the regulatory mechanisms of sugar metabolism in maize stalks. Transcription factors can bind to the promoter regions of target genes, thus regulating gene expression (Jiang et al., 2022). During sugar metabolism in maize stalks, the interactions between transcription factors and sugar metabolism-related genes may precisely regulate the expression levels of sugar metabolism-related genes in response to changes in planting density and growth stage, thus adapting to the needs of different growth stages and environmental conditions of the plant. Shi et al., 2024 suggest that certain transcription factors may promote sucrose synthesis and accumulation by interacting with sugar metabolism genes under high-density planting conditions, thus enhancing the plant’s adaptability to environmental stress. This complex regulatory network represents a finely tuned regulatory mechanism evolved over long-term plant evolution, which is crucial for maintaining sugar metabolism balance in the plant and ensuring normal growth and development.

The synergistic response between metabolites and gene modules is a core feature of regulatory networks. WGCNA analysis showed that 8,154 differentially expressed genes in the MEchocolate3 module were significantly negatively correlated with arabinose and galactose, while the MEgrey60 and MEantiquewhite1 modules were positively correlated with these two monosaccharides. Arabinose and galactose are key components of cell wall arabinogalactans (Konishi et al., 2007) and their metabolic balance may be regulated by glycosyltransferase genes in the MEchocolate3 module. The glycosyltransferase family genes enriched in this module may inhibit the incorporation of arabinose into the cell wall, leading to reduced accumulation of free monosaccharides, which aligns with the requirement for thickening the cell wall of stalks under high density conditions to enhance mechanical strength (Laursen et al., 2018; Gall et al., 2015). Additionally, the MEmediumpurple1 module is positively correlated with glucuronic acid (Glc-UA), which is a precursor for pectin synthesis, suggesting that this module may influence pectin metabolism by regulating UDP-glucose dehydrogenase genes (such as *UGD1*), thus participating in stem strength regulation (Fan et al., 2013; Konstantinos, 2021; Mohnen, 2008).

Hormone signaling pathways act as the “signaling hub” of the network and are deeply coupled with sugar metabolism. KEGG enrichment analysis showed that the plant hormone signal transduction pathway was significantly enriched in all treatments and shared a large number of overlapping genes with the starch-sucrose metabolism pathway. Auxin may influence sucrose distribution by regulating the polar localization of *SUT1*. Studies in tomatoes have confirmed that auxin response factors (ARFs) can directly bind to the *SUT*1 promoter (Yuan et al., 2019). In this experiment, the increase in auxin content in M2 stems at high density was synchronized with the upregulation of the *SPS1* gene. M1 and M2 exhibited significant differences in sugar metabolism gene expression. *IVR1/2* in M2 was upregulated with the growth stage at density D2, while no significant change was observed in M1. Increased IVR activity accelerates sucrose breakdown into hexoses, providing energy for the rapid growth of M2 at high density.

In summary, the regulatory network of sugar metabolism in maize stalk is based on “metabolite-gene modules” as the basic unit, achieving cross-pathway regulation through hormone signaling. Key genes have been identified and their interactions have been analyzed to preliminarily construct a regulatory network of sugar metabolism, providing genetic resources for further exploration of sugar metabolism mechanisms in maize stalks at different planting densities. By studying the dynamic changes and fine-tuning mechanisms of this regulatory network, it will help to comprehensively elucidate the adaptive mechanisms of maize at different planting densities, providing a theoretical basis for enhancing maize’s resistance to lodging.

## 5 Conclusion

This experiment determined the differences in gene expression, structural substances of the stalk, and sucrose accumulation in the third basal internodes of maize stalks at different developmental stages and different planting densities. Using targeted metabolomics and transcriptomics technologies, it was found that glucose is the main sugar metabolite in the internodes of the stem base in the VT-R2 stage, accounting for 60%–70%, while galacturonic acid content is the lowest. A total of 39,757 differentially expressed genes were identified, whose expression patterns were influenced by variety, density, and growth stage, and were enriched in pathways such as hormone signaling transduction, starch metabolism, and sucrose metabolism. Through WGCNA analysis, modules related to monosaccharide metabolites were revealed, and 12 sugar metabolism-related differentially expressed genes were identified. From them, 10 candidate transcription factors potentially involved in sugar metabolism in maize stalks at different densities and growth stages were screened, and a regulatory network of sugar metabolism was constructed. The analysis of this network not only provides a theoretical basis for studying the sugar metabolism response mechanism of maize stalks at different planting densities, but also indirectly provides genetic resources for research on the lodging resistance mechanism of maize stalks. In the future, through further research into the dynamic changes of this regulatory network, it will help to comprehensively understand the adaptive mechanisms of maize at different planting densities, providing a more solid theoretical basis for achieving the goal of increasing maize production through dense planting.

## Data Availability

The datasets presented in this study can be found in online repositories. The names of the repository/repositories and accession number(s) can be found in the article/**Supplementary Material**.

## Supplementary material

Supplementary Table S1 Statistics of transcriptome sequencing data for the 3rd basal internode of maize under different planting densities and developmental stages.

Supplementary Table S2 Statistics of quality evaluation for transcriptome sequencing of maize stalks at different developmental stages under different planting densities.

Supplementary Table S3 Basic information and transcription factor family classification of candidate genes.

Supplementary Figure S1 Boxplot of log_10_ (FPKM) distribution for transcriptome sequencing samples. M1 and M2 represent B12 and J1590, respectively; D1 and D2 represent 105,000 plants ha^-1^ and 135,000 plants ha^-1^, respectively; VT and R2 represent the tasseling stage and grain filling stage, respectively. Z1 and Z2 represent M1 at the VT stage under D1 and D2 densities, respectively; Z3 and Z4 represent M1 at the R2 stage under D1 and D2 densities, respectively; X1 and X2 represent M2 at the VT stage under D1 and D2 densities, respectively; X3 and X4 represent M2 at the R2 stage under D1 and D2 densities, respectively.
