# Supplementary material for: Transcriptome and targeted metabolome analysis reveals sugar metabolism regulation in maize stalks at different densities
Source: Front Plant Sci. 2026 May 8;17:1784881. doi: 10.3389/fpls.2026.1784881 (PMC13194476; doi:10.3389/fpls.2026.1784881)
Supplement: Supplementary file 1 [file SupplementaryFile1.pdf]

Supplementary material

Table S1. Statistics of transcriptome sequencing data for the 3rd basal internode of maize under different planting densities and developmental stages

SampleID	Clean reads pairs	Clean base(G)	Q20(%)	Q30(%)	GC(%)
Z1-1	56694404	8.41	99.26	96.86	51.76
Z1-2	47937640	7.11	99.24	96.79	52.49
Z1-3	52275946	7.75	99.18	96.58	52.16
Z2-1	50348644	7.48	99.32	97.13	52.86
Z2-2	47795952	7.09	99.25	96.86	52.71
Z2-3	55445910	8.23	99.33	97.16	52.97
Z3-1	52584806	7.80	99.27	96.93	52.60
Z3-2	47355682	7.01	99.02	96.02	52.53
Z3-3	45862486	6.80	99.26	96.87	52.57
Z4-1	48925678	7.26	99.22	96.70	53.00
Z4-2	54087254	8.02	99.19	96.61	53.03
Z4-3	51393380	7.62	99.25	96.88	53.17
X1-1	41645278	6.18	99.28	97.00	53.13
X1-2	43861938	6.51	99.27	97.51	53.59
X1-3	45425040	6.74	99.28	97.57	53.82
X2-1	47900834	7.11	99.30	97.05	53.42
X2-2	48468482	7.19	99.23	96.76	53.25
X2-3	51584856	7.65	99.27	96.94	53.37
X3-1	49287254	7.31	99.27	96.93	52.80
X3-2	48388822	7.18	99.29	97.00	52.50
X3-3	49581294	7.35	99.24	96.79	52.61
X4-1	53889508	8.00	99.24	96.83	52.04
X4-2	57457860	8.53	99.33	97.14	52.28
X4-3	55910762	8.31	99.32	97.10	51.02

Note: Z1 and Z2 represent M1 at the VT stage under D1 and D2 planting densities, respectively; Z3 and Z4 represent M1 at the R2 stage under D1 and D2 planting densities, respectively; X1 and X2 represent M2 at the VT stage under D1 and D2 planting densities, respectively; X3 and X4 represent M2 at the R2 stage under D1 and D2 planting densities, respectively.

Table S2. Statistics of quality evaluation for transcriptome sequencing of maize stalks at different developmental stages under different planting densities

Sample	Total reads after filtered	Total mappedr eads	Unique mapped reads	Multiple mapped reads
Z1-1	56694404	53059738 (93.59%)	50819950 (89.64%)	2239788 (3.95%)
Z1-2	47937640	45227156 (94.35%)	43343996 (90.42%)	1883160 (3.93%)

Z1-3	52275946	49493594 (94.68%)	47396990 (90.67%)	2096604 (4.01%)
Z2-1	50348644	47920458 (95.18%)	45974774 (91.31%)	1945684 (3.86%)
Z2-2	47795952	45203654 (94.58%)	43380994 (90.76%)	1822660 (3.81%)
Z2-3	55445910	53010928 (95.61%)	50859916 (91.73%)	2151012 (3.88%)
Z3-1	52584806	49788680 (94.68%)	47737638 (90.78%)	2051042 (3.90%)
Z3-2	47355682	44437298 (93.84%)	42631314 (90.02%)	1805984 (3.81%)
Z3-3	45862486	43557630 (94.97%)	41756778 (91.05%)	1800852 (3.93%)
Z4-1	48925678	46403982 (94.85%)	44422278 (90.80%)	1981704 (4.05%)
Z4-2	54087254	51182216 (94.63%)	49021748 (90.63%)	2160468 (3.99%)
Z4-3	51393380	49093058 (95.52%)	47013114 (91.48%)	2079944 (4.05%)
X1-1	41645278	39330378 (94.44%)	37839704 (90.86%)	1490674 (3.58%)
X1-2	43861938	41408516 (94.41%)	39839890 (90.83%)	1568626 (3.58%)
X1-3	45425040	43063030 (94.80%)	41429216 (91.20%)	1633814 (3.60%)
X2-1	47900834	45384086 (94.75%)	43377862 (90.56%)	2006224 (4.19%)
X2-2	48468482	45056764 (92.96%)	43058492 (88.84%)	1998272 (4.12%)
X2-3	51584856	48818478 (94.64%)	46637684 (90.41%)	2180794 (4.23%)
X3-1	49287254	47206798 (95.78%)	45407224 (92.13%)	1799574 (3.65%)
X3-2	48388822	46258386 (95.60%)	44494276 (91.95%)	1764110 (3.65%)
X3-3	49581294	47126340 (95.05%)	45341754 (91.45%)	1784586 (3.60%)
X4-1	53889508	50766776 (94.21%)	48613178 (90.21%)	2153598 (4.00%)
X4-2	57457860	54531266 (94.91%)	52218178 (90.88%)	2313088 (4.03%)
X4-3	55910762	53117032 (95.00%)	50908988 (91.05%)	2208044 (3.95%)

Note: Table S2. Z1 and Z2 represent M1 at the VT stage under D1 and D2 densities, respectively; Z3 and Z4 represent M1 at the R2 stage under D1 and D2 densities, respectively; X1 and X2 represent M2 at the VT stage under D1 and D2 densities, respectively; X3 and X4 represent M2 at the R2 stage under D1 and D2 densities, respectively.

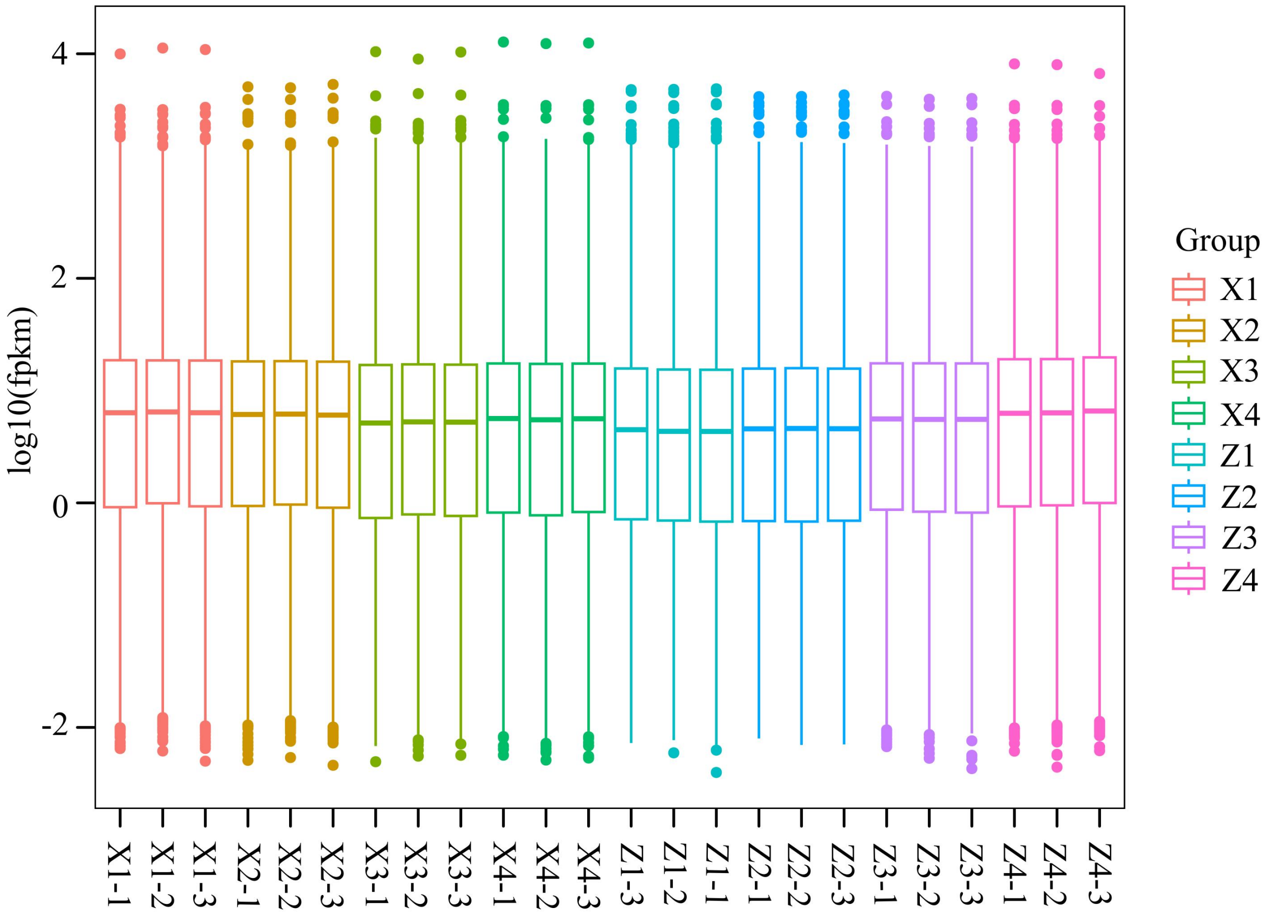

Figure. S1. Boxplot of log_{10}(FPKM) distribution for transcriptome sequencing samples. M1 and M2 represent B12 and J1590, respectively; D1 and D2 represent 105,000 plants ha^{-1} and 135,000 plants ha^{-1}, respectively; VT and R2 represent the tasseling stage and grain filling stage, respectively. Z1 and Z2 represent M1 at the VT stage under D1 and D2 densities, respectively; Z3 and Z4 represent M1 at the R2 stage under D1 and D2 densities, respectively; X1 and X2 represent M2 at the VT stage under D1 and D2 densities, respectively; X3 and X4 represent M2 at the R2 stage under D1 and D2 densities, respectively.

Table S3 Basic information and transcription factor family classification of candidate genes

GeneID	Gene symbol	TF	Protein ID
Zm00001eb169740	*LOC103652922*	bHLH	XP_008678136.1
Zm00001eb015470	*LOC100276044*	bHLH	NP_001350219.1
Zm00001eb075530	*LOC100383472*	bHLH	XP_008668436.1

Zm00001eb272420	*LOC100285948*	HD-ZIP	XP_002439007.1
Zm00001eb364620	*LOC542711*	Glycosyltransferase	NP_001105694.1
Zm00001eb368380	*LOC100285126*	B3	NP_001151493.1
Zm00001eb299100	*LOC103631909*	WRKY	XP_008651978.1
Zm00001eb057060	*LOC100277055*	GRAS	NP_001144195.2
Zm00001eb007000	*LOC100216971*	ERF	NP_001136823.1
Zm00001eb234400	*LOC103626734*	ERF	NP_001136823.1
